# Risk and Outcomes of Secondary Cancer Among Lung Cancer Survivors After Definitive Treatment

**DOI:** 10.1001/jamanetworkopen.2025.47831

**Published:** 2025-12-09

**Authors:** Matthew T. McMillan, Orly Yariv, Sana Raoof, Jamie E. Chaft, Bernard J. Park, David R. Jones, Narek Shaverdian, Julianne Ruggiero, Unnati Jackson, Bob T. Li, Puneeth Iyengar, Daniel R. Gomez

**Affiliations:** 1 Department of Radiation Oncology, Memorial Sloan-Kettering Cancer Center, New York, New York; 2 Department of Medicine, Memorial Sloan-Kettering Cancer Center, New York, New York; 3 Department of Surgery, Memorial Sloan-Kettering Cancer Center, New York, New York; 4 Division of Nursing, Memorial Sloan-Kettering Cancer Center, New York, New York

## Abstract

**Importance:**

Second primary cancers are an important cause of morbidity, mortality, and resource use among lung cancer survivors, yet their risk relative to recurrence and their determinants have been incompletely defined.

**Objectives:**

To quantify the competing risks of recurrence, intrathoracic new cancer (locoregional or distant recurrences confined to the thorax and/or second primary lung cancers), and non-lung secondary cancers (NLSCs) after curative-intent local therapy for non–small cell lung cancer (NSCLC) and to identify clinical factors associated with NLSC.

**Design, Setting, and Participants:**

This cohort study included consecutive stage I to III NSCLC survivors treated with definitive local therapy and evaluated in a dedicated survivorship clinic at a single high-volume academic cancer center from January to May 2019. Follow-up was measured from completion of local therapy; eligible participants were disease-free 12 months or longer following curative-intent surgery or radiotherapy. Data were analyzed in October 2025.

**Exposures:**

Definitive local therapy (surgery or radiotherapy) for NSCLC followed by survivorship care.

**Main Outcomes and Measures:**

The outcomes were incidence and timing of recurrence, NLSC, intrathoracic new cancer, extrathoracic cancer, or death measured using nonparametric cumulative incidence functions (CIFs), with death as a competing event, and multivariable Fine-Gray and cause-specific Cox models for NLSC. Overall survival (OS) was estimated using the Kaplan-Meier method.

**Results:**

Among 496 survivors (58.5% female [290]; median [IQR] age, 69.1 [62.8-74.3] years), the median (IQR) follow-up was 71.6 (57.7-84.8) months. Of these patients, 367 (74.0%) were former smokers; the index cancer was adenocarcinoma in 372 (75.0%); 337 (67.9%) had stage I disease, 72 (14.5%) had stage II, and 87 (17.5%) had stage III. Recurrence occurred in 67 of 496 patients (13.5%). Secondary cancers developed in 116 of 496 patients (23.4%), including a new primary lung cancer in 77 of 496 patients (15.5%) and NLSC in 39 of 496 patients (7.9%). Median (IQR) time to diagnosis of an NLSC was 52.3 (35.9-65.6) months, and CIFs (competing risks) at 5 years (death and other first events treated as competing risks) were 11.5% for recurrence, 5.6% for NLSC, 16.8% for intrathoracic new cancer, and 10.4% for extrathoracic cancer. In Fine-Gray models, a hereditary syndrome and/or pathogenic germline variant was associated with higher risk for NLSC (subdistribution hazard ratio [SHR], 10.76; 95% CI, 4.62-25.06; *P* < .001), whereas pack-years (per 10) were not associated with higher risk (SHR, 1.00; 95% CI, 0.97-1.03; *P* = .85). Cause-specific Cox results were concordant (HR, 8.32; 95% CI, 3.14-22.02; *P* < .001).

**Conclusions and Relevance:**

In this cohort study of NSCLC survivors, the risk of NLSC was clinically meaningful and distinct from intrathoracic new cancers. Genetic predisposition correlated with NLSC risk and should inform survivorship care pathways.

## Key Points

**Question:**

Among non–small cell lung cancer (NSCLC) survivors who underwent curative-intent local therapy, what is the incidence and timing of non-lung secondary cancers (NLSCs) vs intrathoracic new cancers (locoregional or distant recurrences confined to the thorax and/or second primary lung cancers), and which clinical factors are associated with NLSCs?

**Findings:**

In this cohort study of 496 NSCLC survivors, 5-year cumulative incidence was 11.5% for recurrence and 5.6% for NLSC. Intrathoracic new cancer incidence was 16.8% vs 10.4% for extrathoracic cancer at 5 years.

**Meaning:**

These findings suggest the risk of NLSC is clinically meaningful and concentrated in genetically predisposed survivors, supporting risk-adapted surveillance beyond the chest.

## Teaser

This cohort study assesses the incidence of secondary cancers and recurrence of the index non–small cell lung cancer among patients with non–small cell lung cancer treated with curative intent who were disease-free for 12 months or longer.

## Introduction

In the past decade, longer survival times for patients with non–small cell lung cancer (NSCLC) have been achieved through treatment improvements,^1,2,3^ broader access to care,^4^ earlier detection,^5,6^ and advances in staging.^7^ As a result, the population of lung cancer survivors is expanding and is estimated to exceed 20 million by 2026.^8^ Survivors of NSCLC remain at risk for both disease recurrence and secondary cancers, particularly second primary lung cancers,^9^ which often present as early-stage disease and may be curable with surgery. However, data on secondary cancer incidence among NSCLC survivors are limited. Among early-stage survivors, recurrence rates range from 10% to 38%, and the risk of a second primary cancer is estimated at 1% to 2% per year.^10,11^

While routine surveillance with chest computed tomography (CT) scans detects most intrathoracic recurrences and second primary lung cancers, non-lung secondary cancers (NLSCs) are often identified incidentally or through symptom-driven evaluations. Their true incidence, timing, and outcomes remain poorly characterized. We evaluated a cohort of NSCLC survivors seen in a dedicated survivorship clinic to assess the incidence, timing, and outcomes of secondary cancers. We hypothesized that the risk of secondary cancers—including NLSCs not captured by routine chest imaging—would remain elevated over time and play a role in late morbidity and mortality.

## Methods

### Study Design, Oversight, and Reporting

This single-center retrospective cohort study was approved by the Institutional Review Board of Memorial Sloan Kettering Cancer Center with a waiver of informed consent due to minimal risk and protection of patient privacy. We followed the Strengthening the Reporting of Observational Studies in Epidemiology (STROBE) reporting guideline for cohort studies.

### Setting, Cohort Accrual, and Eligibility

We identified consecutive NSCLC survivors seen in a dedicated thoracic cancer survivorship clinic at a single high-volume academic cancer center between January and May 2019. The survivorship roster (a population typically 2 years or more removed from having received definitive local therapy, with discretionary earlier referrals) was queried for all visits within the accrual window. Of 530 screened records, 34 were excluded prior to analysis due to no evidence of disease less than 12 months after local therapy (n = 28), small cell lung cancer (n = 4), sarcoma (n = 1), or metastatic disease at diagnosis (n = 1), yielding 496 eligible patients for the analytic cohort (eFigure in Supplement 1). Inclusion criteria were: (1) stage I to III NSCLC; (2) completion of curative-intent local therapy (surgery and/or radiotherapy); and (3) no evidence of disease for 12 months or longer at cohort entry. Exclusion criteria were small cell histology, stage IV disease at diagnosis, incomplete local therapy, or recurrence within 12 months.

### Survivorship Care 

Institutional practice is to transition patients who are disease-free to survivorship care approximately 24 months after completion of local therapy; final timing is at the treatment team’s discretion. Survivors are followed by advanced practice professionals in thoracic surgery with multidisciplinary coordination. Routine thoracic surveillance consists of a baseline chest CT approximately 3 months after local therapy, then chest CT every 3 to 4 months in years 1 and 2, every 6 months in years 3 and 4, and annually thereafter; positron emission tomography/CT, brain magnetic resonance imaging, or other imaging is obtained for symptoms or abnormal findings. Guideline-concordant, age-appropriate population screening for breast, colorectal, or prostate cancer is coordinated with primary care.

### Data Sources and Variables

Demographics (age, sex assigned at birth), race and ethnicity, tobacco exposure, comorbidities, prior cancer history, index tumor histology, stage, treatment modality, and hereditary cancer information (clinical features and/or pathogenic germline variants) were abstracted from the electronic health record. Race and ethnicity data were collected to characterize the sample and not analyzed further. Race and ethnicity were self-reported using predefined options at intake. Ethnicity was categorized as either Hispanic or non-Hispanic. Race categories included American Indian or Native American, Asian, Black or African American, White, and declined or unknown.

For analysis, disease stage was harmonized to stage groups I, II, or III (stage II, IIA, or 2 were considered stage group II). Tobacco exposure was modeled as per 10 pack-years rather than categorical smoking status given that less than 1% of patients had no smoking history. When available, we also captured for each NLSC the mode of detection—symptom driven, screening, or incidental—and whether age-appropriate population screening was up to date at diagnosis.

### Outcomes and Definitions 

Time zero was the date of completion of curative-intent local therapy for the index NSCLC. Outcomes were incidence and timing of recurrence, NLSC, intrathoracic new cancer, extrathoracic cancer, or death.

Second primary lung cancers were distinguished from recurrence using molecular profiling when available; otherwise, Martini-Melamed^12^ criteria were applied. For squamous cell carcinomas (SCCs), the primary site was adjudicated using clinical context, imaging distribution, and pathology; when comprehensive profiling or human papillomavirus p16 status was unavailable, anatomic–pathologic criteria were used, acknowledging residual uncertainty in distinguishing metachronous head and neck SCCs from a second primary lung SCC.

### Statistical Analysis

OS was estimated using the Kaplan-Meier method with Greenwood 95% CIs. Cumulative incidence functions (CIFs) were estimated for competing end points using the Fine-Gray method. The primary multivariable model evaluated factors associated with the incidence of NLSC using Fine-Gray subdistribution hazards (SHRs) including age, sex, pack-years, *American Joint Committee on Cancer Staging Manual, 8th Edition* stage group (I, II, or III), index NSCLC histology, prior cancer (excluding nonmelanoma skin cancer), treatment modality (surgery and/or radiotherapy), hereditary syndrome, and/or germline pathogenic variant. As a sensitivity analysis, we fit cause-specific Cox models for NLSC with the same covariates, treating other events as censored. Complete-case analyses were performed, with missing data tabulated for all baseline covariates. Effect sizes are presented as SHRs or cause-specific hazard ratios (HRs) with 95% CIs; 2-sided *P* values < .05 were considered statistically significant. Analyses were conducted in R, version 2025.05.01 (R Project for Statistical Computing).

## Results

### Cohort and Follow-Up

Four hundred ninety-six NSCLC survivors (290 females [58.5%], 206 males [41.5%]; median [IQR] age, 69.1 [62.8-74.3] years) were included in the analysis. Of these patients, 337 (67.9%) had stage I, 72 (14.5%) had stage II, and 87 (17.5%) had stage III cancer, 367 (74.0%) were former smokers, and 372 (75.0%) had adenocarcinoma. Twelve patients were Hispanic (2.4%), 481 non-Hispanic (97.0%), 2 were American Indian or Native American (0.4%), 15 were Asian (3.0%), 12 were Black or African American (2.4%), 454 were White (91.5%), and 13 were of unknown race or declined to answer (2.6%). Median (IQR) follow-up from completion of local therapy was 71.6 (57.7-84.8) months. Baseline characteristics are summarized in Table 1; participant inclusion flow is shown in the eFigure in Supplement 1. Missing baseline data were minimal.

**Table 1.** Baseline Characteristics of the Survivorship Cohort

Characteristic	No. (%) (N = 496)^a^
Age at local therapy completion, median (IQR), y	69.1 (62.8-74.3)
Sex at birth	
Female	290 (58.5)
Male	206 (41.5)
Ethnicity	
Hispanic (not otherwise specified)	12 (2.4)
Non-Hispanic	481 (97.0)
Unknown	3 (0.6)
Race	
American Indian or Native American	2 (0.4)
Asian	15 (3.0)
Black or African American	12 (2.4)
White	454 (91.5)
Declined or unknown	13 (2.6)
Smoking status	
Never	2 (0.4)
Former	367 (74.0)
Current	127 (25.6)
Pack-years, median (IQR)	35.0 (20.0-50.0)
Alcohol use	
None or past	190 (38.3)
Social	169 (34.1)
At least weekly	137 (27.6)
Index cancer characteristics	
Index non–small cell lung cancer histology	
Adenocarcinoma	372 (75.0)
Squamous cell carcinoma	88 (17.7)
Other^b^	36 (7.3)
Cancer history	
Prior cancer (including nonmelanoma skin cancer)	205 (41.3)
Prior cancer (excluding nonmelanoma skin cancer)	177 (35.7)
Comorbidities	
Chronic obstructive pulmonary disease	191 (38.5)
Autoimmune disease	60 (12.1)
Hypertension	326 (65.7)
Coronary artery disease	127 (25.6)
Diabetes	100 (20.2)
Chronic pancreatitis	5 (1.0)
Cirrhosis	3 (0.6)
Family history	
Any family history of cancer (first- or second-degree relative)^c^	
No	149 (30.0)
Yes	342 (69.0)
Unknown (adopted)	5 (1.0)
First-degree family history of any cancer	
No	183 (36.9)
Yes	308 (62.1)
Unknown (adopted)	5 (1.0)
First-degree family history of lung cancer	
No	382 (77.0)
Yes	109 (22.0)
Unknown (adopted)	5 (1.0)
Number of affected first-degree relatives	
0	183 (36.9)
1	183 (36.9)
2	92 (18.5)
≥3	33 (6.7)
Unknown (adopted)	5 (1.0)
Documented early-onset cancer in first-degree relatives^d^	
No	473 (95.4)
Yes	18 (3.6)
Unknown (adopted)	5 (1.0)
Hereditary cancer syndrome or pathogenic germline variant^e^	12 (2.4)
Treatment	
Definitive local therapy	
Surgery	446 (89.9)
Radiotherapy	50 (10.1)
Follow-up, median (IQR), mo^f^	71.6 (57.7-84.8)

^a^ Percentages may not total 100 due to rounding.

^b^ Other includes adenosquamous carcinoma, sarcomatoid carcinoma, basaloid squamous cell carcinoma, and non–small cell lung cancer not otherwise specified.

^c^ First-degree relatives include parents, siblings, and children.

^d^ Diagnosis at age younger than 50 years.

^e^ Documentation of a clinical hereditary cancer syndrome and/or a pathogenic germline variant by clinical testing.

^f^ Measured from completion of definitive local therapy to last clinic contact or death.

### Distribution of First Events and Competing Risks

**Figure 1.**
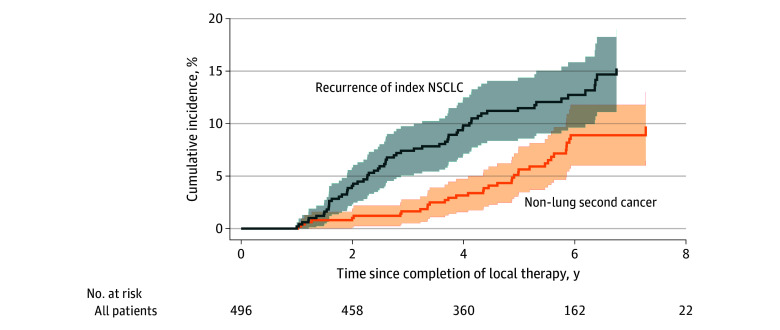
Competing-Risk Cumulative Incidence Functions for Recurrence of the Index Non–Small Cell Lung Cancer vs Non-Lung Secondary Cancer Shaded areas represent 95% CIs.

The first observed outcome was recurrence in 63 patients (12.7%), NLSC in 36 (7.3%), a new second primary lung cancer in 76 (15.3%), and death before any other event in 34 (6.9%); 287 patients (57.9%) were censored (eTable 1 in Supplement 1). According to Fine-Gray estimation from completion of local therapy, cumulative incidence of recurrence was 4.1% at 2 years, 7.4% at 3 years, 9.8% at 4 years, and 11.5% at 5 years (Figure 1; eTable 2 in Supplement 1). The corresponding NLSC cumulative incidences were 1.0%, 1.6%, 3.2%, and 5.6% at 2, 3, 4, and 5 years, respectively.

Sixty-seven patients (13.5%) experienced disease recurrence at any time after local therapy; the median (IQR) time to first documented recurrence was 31.5 (21.8-49.7) months. Patterns of first documented recurrence were local in 23 patients (34.3%), regional in 15 (22.4%), and distant with or without locoregional in 29 (43.3%).

### Cumulative Incidence With Competing Risks

**Figure 2.**
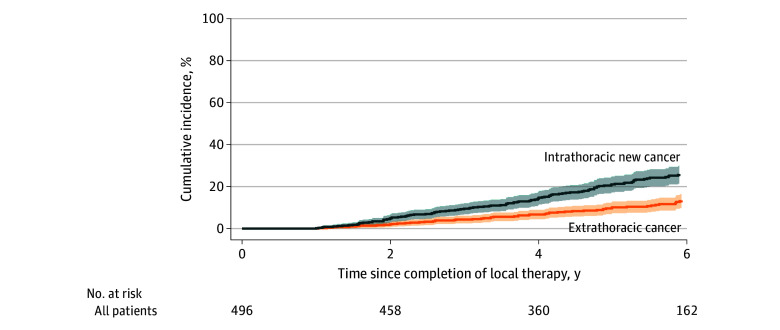
Competing-Risk Cumulative Incidence Functions for Intrathoracic vs Extrathoracic New Cancer Shaded areas represent 95% CIs.

Using a first-event framework with death and other event types treated as competing risks, the 2-, 3-, 4-, and 5-year CIFs of recurrence were 4.1%, 7.4%, 9.8%, and 11.5%, respectively (Figure 1; eTable 2 in Supplement 1). Over the same time points, the CIFs of NLSCs were 1.0%, 1.6%, 3.2%, and 5.6%, respectively. The 2-, 3-, 4-, and 5-year CIFs of intrathoracic new cancer were 4.3%, 8.0%, 12.0%, and 16.8%, respectively. The 2-, 3-, 4-, and 5-year CIFs of extrathoracic cancer were 1.8%, 4.1%, 6.7%, and 10.4%, respectively (Figure 2; eTable 2 in Supplement 1).

### Characteristics and Detection of Secondary Cancers

At the end of the study period, 116 of 496 survivors (23.4%) had developed a secondary cancer: 77 (15.5%) had new second primary lung cancers (median [IQR], 56.1 [37.8-76.5] months from local therapy) and 39 (7.9%) had NLSCs (median [IQR], 52.3 [35.9-65.6] months from local therapy). The most frequent NLSCs were breast (6 [15.4%]), prostate (5 [12.8%]), pancreatic (5 [12.8%]), and head and neck cancers (5 [12.8%]); 8 (20.5%) presented with metastatic disease. Of the 39 NLSCs, 27 (69.2%) were symptom detected and 12 (30.8%) were incidental; none were first identified through population screening. Among the 14 patients who had NLSCs with established screening recommendations (breast [n = 6], colorectal [n = 3], and prostate [n = 5]), all patients were up to date with age-appropriate screening at the time of diagnosis (eTable 3 in Supplement 1).

### Clinicopathologic Features and Management

Second primary lung cancers were predominantly adenocarcinoma (59 [76.6%]), followed by squamous cell carcinoma (11 [14.3%]), with small-cell histology uncommon (4 [5.2%]). The median (IQR) interval from local therapy to second primary lung cancer was 56.1 (37.8-76.5) months. NLSCs spanned a broad spectrum, with stage at diagnosis distributed as in situ in 3 (7.7%), stage 1 in 17 (43.6%), stage 2 in 3 (7.7%), stage 3 in 8 (20.5%), and stage 4 in 8 (20.5%). Initial management of NLSC was most often surgical (24 [61.5%]); systemic therapy was less common (chemotherapy, 4 [10.3%]; hormonal, 3 [7.7%]; and biologic, 1 [2.6%]) and 3 NLSCs (7.7%) received no cancer-directed treatment. These patterns align with the observed cumulative incidence profiles (Figure 2).

### OS

**Figure 3.**
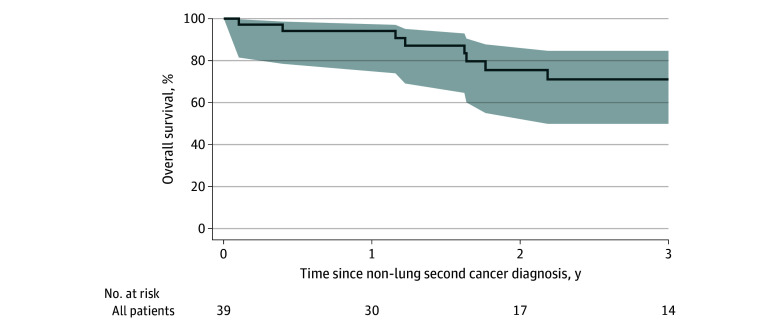
Overall Survival After Being Diagnosed With a Non-Lung Secondary Cancer Shaded areas represent 95% CIs.

Median whole cohort OS from completion of local therapy was not reached. Point estimates at 2, 3, and 5 years were 99.8% (95% CI 98.6%-100%), 97.7% (95% CI 95.9%-98.7%), and 93.2% (95% CI 90.3%-95.2%), respectively. Among patients with a NLSC, 2-year OS from NLSC diagnosis was 73.3% (95% CI, 51.6%-86.5%) (Figure 3).

### Multivariable Associations With NLSC

In prespecified models, pack-years were modeled per 10–pack-year increment, with stage as I, II, or III. In multivariable Fine-Gray models for NLSC (event of interest; competing events: recurrence, second primary lung cancer, and death), hereditary cancer syndrome and/or a pathogenic germline variant was associated with a higher SHR (10.76; 95% CI, 4.62-25.06; *P* < .001), whereas pack-years (per 10) were not associated with higher risk (SHR, 1.00; 95% CI 0.97-1.03, *P* = .85) (Table 2). In a prespecified cause-specific Cox sensitivity analysis, the association for pack-years smoking (per 10 pack-years) was borderline inverse (HR 0.88; 95% CI, 0.77-1.00, *P* = .05). Results were directionally consistent in cause-specific Cox models as well, including hereditary cancer syndrome and/or a pathogenic germline variant being associated with higher risk for NLSC (HR, 8.32; 95% CI, 3.14-22.02; *P* < .001) (eTable 4 in Supplement 1).

**Table 2.** Multivariable Fine-Gray Subdistribution Model for Non-Lung Secondary Cancers^a^^,^^b^

Characteristic	SHR (95% CI)^c^	*P* value
Age^d^	1.02 (0.97-1.06)	.36
Sex		
Male	1.37 (0.70-2.69)	.39
Female	1 [Reference]	NA
Pack-years smoking^d^	1.00 (0.97-1.03)	.85
Hereditary syndrome and/or pathogenic germline variant^e^	10.76 (4.62-25.06)	<.001
AJCC stage group		
I	1 [Reference]	
II	0.79 (0.27-2.28)	.77
III	0.39 (0.09-1.62)	.15
Index NSCLC histology		
Adenocarcinoma	1 [Reference]	
Squamous cell carcinoma	1.46 (0.64-3.33)	.49
Other	2.56 (0.92-7.13)	.11
Prior cancer		
Yes	0.91 (0.44-1.87)	.87
No	1 [Reference]	NA
Previous treatment		
RT for local therapy	1.22 (0.46-3.21)	.54
Surgery	1 [Reference]	NA

Abbreviations: AJCC, American Joint Committee on Cancer; NSCLC, non–small cell lung cancer; RT, radiotherapy; SHR, subdistribution hazard ratio.

^a^ Event of interest was non-lung secondary cancers; competing risks were intrathoracic recurrence or a second primary lung cancer, and death.

^b^ Model fitted on complete cases without imputation.

^c^ SHR greater than 1 indicates higher cumulative incidence of non-lung secondary cancers in the presence of competing risks. SHRs estimate associations between baseline factors and the cumulative incidence of non-lung secondary cancers after curative-intent local therapy for stage I to III non–small cell lung cancer; intrathoracic recurrence, a second primary lung cancer, and death were treated as competing risks.

^d^ Continuous covariates: age per 1 year; pack-years scaled per 10.

^e^ Documentation of hereditary cancer syndrome and/or a pathogenic germline variant by clinical testing.

### Sensitivity and Data-Consistency Checks

All event times were validated against event indicators, restricted to more than 0 months, and constrained not to exceed the patient-level follow-up time; deaths were counted only when vital status was coded dead and the follow-up time equaled time to death. First-event distributions under this framework are reported in eTable 1 in Supplement 1 and show expected censoring proportions, supporting the accuracy of the competing-risk analysis.

## Discussion

In this survivorship cohort of patients with stage I to III NSCLC treated with curative-intent local therapy, we found that clinically important new disease accumulated both within and beyond the thorax over a prolonged time, and that NLSCs were enriched among survivors with documented hereditary cancer syndromes and/or pathogenic germline variants. The occurrence of NLSC was characteristically late—often more than 4 years after local therapy—and detection was rarely via population screening. Together, these observations support risk-adapted survivorship pathways that extend vigilance beyond chest-focused imaging and incorporate heritable risk.

Two findings are most actionable. First, approximately 1 in 5 patients with NLSC presented with metastatic disease and carried a poor outcome (median OS approximately 20 months), underscoring the clinical consequence of extrathoracic events. Second, the diagnosis of most NLSCs in our cohort were symptom prompted and nearly one-third were incidental, with no events first identified by population screening—even among cancers with established screening programs. In parallel, nearly 13% of survivors developed extrathoracic cancer or metastases without concurrent thoracic findings, highlighting that thoracic surveillance alone will miss a clinically meaningful fraction of consequential events. These findings argue for risk-aligned strategies—patient education about red-flag symptoms, low-threshold diagnostic evaluation of extrathoracic complaints, and systematic attention to hereditary risk—rather than simply expanding population screening beyond guidelines.

Our results align with and extend prior literature. Earlier studies report that, while recurrence risk declines over time, second primary lung cancers accumulate at an approximately constant rate.^13^ Population-based work similarly shows an elevated burden of subsequent primary cancers among lung cancer survivors.^14^ This study adds the following advances to the literature: (1) direct quantification of competing risks that separates NLSC from intrathoracic new cancer; (2) precise timing showing that NLSC risk persists late into survivorship; and (3) a large, independent association of hereditary predisposition with NLSC in multivariable Fine-Gray models, with concordant cause-specific estimates. Notably, 10–pack-year intervals were not independently associated with NLSC after adjustment, suggesting that inherited susceptibility, rather than smoking intensity alone, may play a role in extrathoracic risk in this setting.

Our findings have immediate practice implications. Guideline-concordant thoracic follow-up should be maintained, but survivorship care should also do the following: (1) standardize documentation of family history and prior cancers; (2) incorporate brief hereditary-risk screening with streamlined referral to genetics when indicated; (3) ensure eligibility for low-dose CT for lung cancer screening; and (4) adopt a low threshold for targeted extrathoracic evaluation when new symptoms arise. Importantly, our data do not support blanket expansion of routine imaging outside the chest; rather, they support risk-adapted, symptom-responsive approaches that can be implemented without excessive testing.

We assembled a consecutive, well-characterized survivorship cohort with long follow-up and used prespecified, transparent time-to-event methods. To minimize outcome misclassification and the appearance of spurious events, we validated all event times against event indicators, constrained event times not to exceed patient-level follow-up, and prioritized disease events over death over administrative censoring for same-month ties. Competing-risk estimates were paired with cause-specific models to show robustness. For second primary cancers vs recurrence, we used molecular profiling when available and otherwise applied Martini-Melamed^12^ criteria with multidisciplinary review, while acknowledging residual uncertainty for squamous cell primaries (eg, head and neck vs lung). Ascertainment bias was considered explicitly; some NLSCs were detected incidentally on nonthoracic imaging, a scenario common in contemporary practice and one that our sensitivity checks were designed to handle. Finally, we tabulated missing data for all baseline covariates and used complete-case analyses as specified.

### Limitations

This single-center study reflects a survivorship clinic that typically transitions disease-free patients approximately 2 years after therapy; because stage II and III disease recurs earlier, this process enriches the cohort for stage I and may limit generalizability. We lacked a reliable denominator of all treated patients with NSCLC during accrual, and therefore, could not estimate the referral or transition rate. Visit and imaging adherence were not uniformly captured in structured fields, potentially influencing detection timing. Despite molecular adjudication and criteria-based review, some site misclassification remains possible for squamous cell carcinomas. Residual confounding is also possible, and we did not evaluate the cost-effectiveness of alternative surveillance strategies.

## Conclusions

In this cohort study of 496 disease-free survivors of stage I to III NSCLC, NLSCs occurred late and were often identified by symptoms or incidentally rather than by screening, while intrathoracic new cancers remained more common overall. Hereditary cancer syndromes or pathogenic germline variants were associated with NLSC. These findings support a pragmatic, risk-adapted survivorship approach that includes maintaining guideline-concordant thoracic imaging, incorporating brief hereditary-risk assessment with genetics referral when indicated, coordinating age-appropriate population screening, and keeping a low threshold to evaluate new extrathoracic symptoms. Prospective, multicenter studies are warranted to validate our findings and to test whether risk-aligned pathways may improve the timeliness of diagnosis, treatment candidacy, and long-term outcomes.

## Notes

Supplement 1. **eFigure.** Participant Flow Diagram **eTable 1.** First Event Distribution **eTable 2.** Cumulative Incidence During the First Five Years After Curative-Intent Local Therapy **eTable 3.** Non-Lung Secondary Cancer (NLSC) Characteristics and Detection Mode **eTable 4.** Cause-Specific Cox Regression for Risk of NLSC

Supplement 2.
Data Sharing Statement

## References

[1] Howlader N, Forjaz G, Mooradian MJ, . The effect of advances in lung-cancer treatment on population mortality. N Engl J Med. 2020;383(7):640-649. doi:10.1056/NEJMoa1916623 32786189 PMC8577315

[2] Muthusamy B, Patil PD, Pennell NA. Perioperative systemic therapy for resectable non-small cell lung cancer. J Natl Compr Canc Netw. 2022;20(8):953-961. doi:10.6004/jnccn.2022.7021 35948038

[3] Jones GS, Baldwin DR. Recent advances in the management of lung cancer. Clin Med (Lond). 2018;18(suppl 2):s41-s46. doi:10.7861/clinmedicine.18-2-s41 29700092 PMC6334032

[4] Liu Y, Colditz GA, Kozower BD, . Association of Medicaid expansion under the Patient Protection and Affordable Care Act with non-small cell lung cancer survival. JAMA Oncol. 2020;6(8):1289-1290. doi:10.1001/jamaoncol.2020.1040 32407435 PMC7226289

[5] Potter AL, Rosenstein AL, Kiang MV, . Association of computed tomography screening with lung cancer stage shift and survival in the United States: quasi-experimental study. BMJ. 2022;376:e069008. doi:10.1136/bmj-2021-069008 35354556 PMC8965744

[6] Kratzer TB, Bandi P, Freedman ND, . Lung cancer statistics, 2023. Cancer. 2024;130(8):1330-1348. doi:10.1002/cncr.35128 38279776

[7] Rami-Porta R, Call S, Dooms C, . Lung cancer staging: a concise update. Eur Respir J. 2018;51(5):1800190. doi:10.1183/13993003.00190-2018 29700105

[8] Miller KD, Siegel RL, Lin CC, . Cancer treatment and survivorship statistics, 2016. CA Cancer J Clin. 2016;66(4):271-289. 27253694 10.3322/caac.21349

[9] Surapaneni R, Singh P, Rajagopalan K, Hageboutros A. Stage I lung cancer survivorship: risk of second malignancies and need for individualized care plan. J Thorac Oncol. 2012;7(8):1252-1256. doi:10.1097/JTO.0b013e3182582a79 22627646

[10] Pepek JM, Chino JP, Marks LB, . How well does the new lung cancer staging system predict for local/regional recurrence after surgery? a comparison of the TNM 6 and 7 systems. J Thorac Oncol. 2011;6(4):757-761. doi:10.1097/JTO.0b013e31821038c0 21325975

[11] Hamaji M, Allen MS, Cassivi SD, . Surgical treatment of metachronous second primary lung cancer after complete resection of non-small cell lung cancer. J Thorac Cardiovasc Surg. 2013;145(3):683-690. doi:10.1016/j.jtcvs.2012.12.051 23414986

[12] Martini N, Melamed MR. Multiple primary lung cancers. J Thorac Cardiovasc Surg. 1975;70(4):606-612. doi:10.1016/S0022-5223(19)40289-4 170482

[13] Lou F, Huang J, Sima CS, Dycoco J, Rusch V, Bach PB. Patterns of recurrence and second primary lung cancer in early-stage lung cancer survivors followed with routine computed tomography surveillance. J Thorac Cardiovasc Surg. 2013;145(1):75-81. doi:10.1016/j.jtcvs.2012.09.030 23127371

[14] Sung H, Hyun N, Leach CR, Yabroff KR, Jemal A. Association of first primary cancer with risk of subsequent primary cancer among survivors of adult-onset cancers in the United States. JAMA. 2020;324(24):2521-2535. doi:10.1001/jama.2020.23130 33351041 PMC7756242

