# Supplementary material for: Risk and Outcomes of Secondary Cancer Among Lung Cancer Survivors After Definitive Treatment
Source: JAMA Netw Open. 2025 Dec 9;8(12):e2547831. doi: 10.1001/jamanetworkopen.2025.47831 (PMC12690424; doi:10.1001/jamanetworkopen.2025.47831)
Supplement: Supplement 1. — eFigure. Participant Flow Diagram eTable 1. First Event Distribution eTable 2. Cumulative Incidence During the First Five Years After Curative-Intent Local Therapy eTable 3. Non-Lung Secondary Cancer (NLSC) Characteristics and Detection Mode eTable 4. Cause-Specific Cox Regression for Risk of NLSC [file jamanetwopen-e2547831-s001.pdf]

Supplemental Online Content

McMillan MT, Yariv O, Raoof S, et al. Risk and outcomes of secondary cancer among lung cancer survivors after definitive treatment. *JAMA Netw Open*. 2025;8(12):e2547831. doi:10.1001/jamanetworkopen.2025.47831

eFigure 1. Participant Flow Diagram
eTable 1. First Event Distribution
eTable 2. Cumulative Incidence During the First Five Years After Curative-Intent Local Therapy
eTable 3. Non-Lung Secondary Cancer (NLSC) Characteristics and Detection Mode
eTable 4. Cause-Specific Cox Regression for Risk of NLSC

This supplemental material has been provided by the authors to give readers additional information about their work.

© 2025 McMillan MT et al. *JAMA Network Open*.

eFigure 1. Participant Flow Diagram

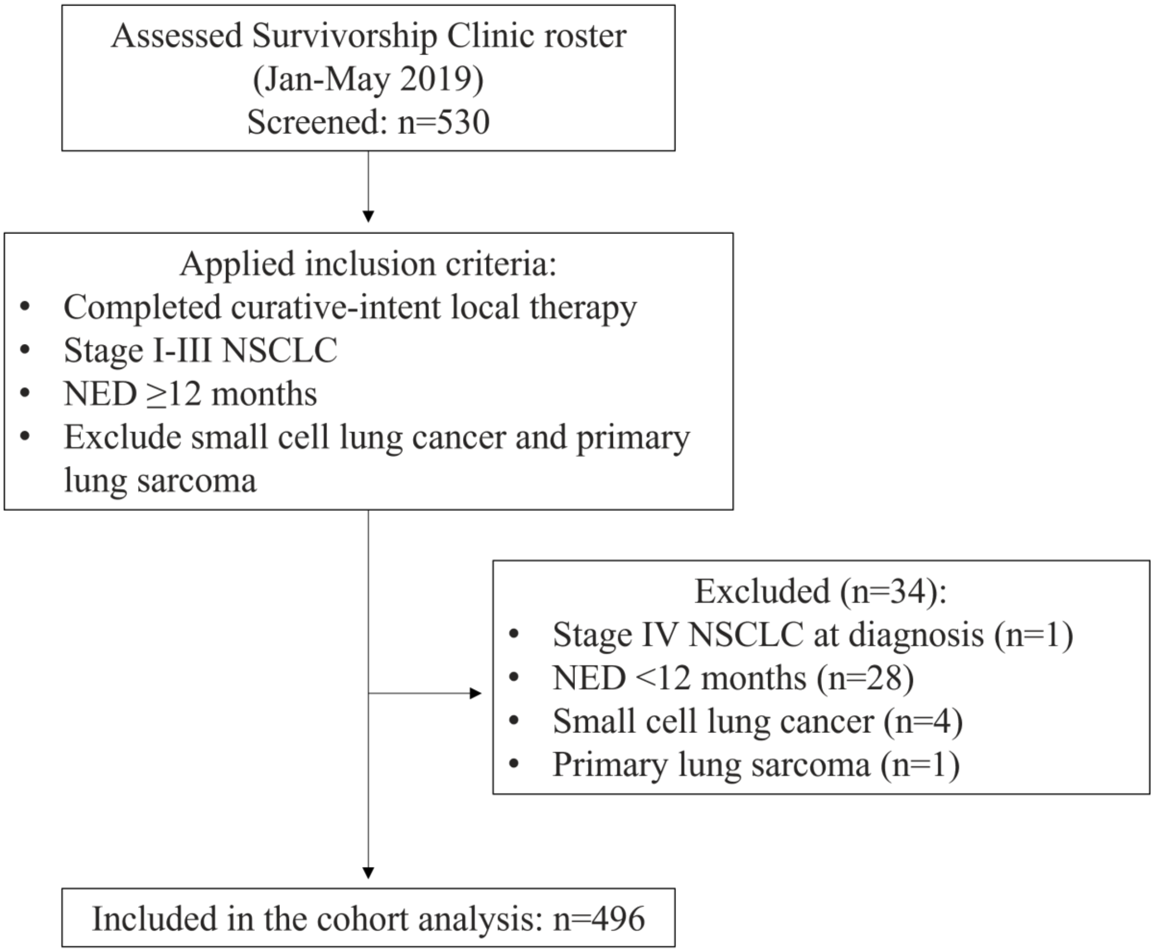

Survivorship clinic roster (January–May 2019) screened n=530; excluded n=34 (not NED ≥12 months, n=28; small-cell lung cancer, n=4; sarcoma, n=1; metastatic at diagnosis, n=1); included in analytic cohort, n=496.

© 2025 McMillan MT et al. *JAMA Network Open*.

eTable 1. First Event Distribution

Legend. Distribution of the earliest observed outcome after completion of curative-intent local therapy among the analytic cohort (N=496).

First Event	n	pct
Censor	287	57.9
Recurrence	63	12.7
NLSC	36	7.3
New lung primary	76	15.3
Death	34	6.9

Abbreviations. NLSC, non-lung secondary cancer.

a. Time zero: date of completion of definitive local therapy (surgery or radiotherapy).
b. “First event” is determined with the following order for ties within the same month: disease event (recurrence, NLSC, or second primary lung cancer) > death > administrative censoring.
c. Percentages are row percentages of the total cohort and may not sum to 100% due to rounding.
d. Censoring reflects alive without any study outcome at last known follow-up.

© 2025 McMillan MT et al. *JAMA Network Open*.

eTable 2. Cumulative Incidence During the First Five Years After Curative-Intent Local Therapy

Legend. Cumulative incidence function (CIF, %) at landmark years from completion of local therapy, estimated under competing risks.

	Cumulative Incidence Function			
Time from local therapy completion, years	Recurrence	NLSM	Intrathoracic cancer	Extrathoracic cancer
2	4.1	1.0	4.3	1.8
3	7.4	1.6	8.0	4.1
4	9.8	3.2	12.0	6.7
5	11.5	5.6	16.8	10.4

Abbreviations. CIF, cumulative incidence function; NLSC, non-lung secondary cancer.

a. Recurrence and NLSC are treated as mutually exclusive event types in the CIF; death is a competing risk.
b. "Intrathoracic cancer" comprises locoregional/distant recurrence confined to the thorax and/or second primary lung cancers.
c. "Extrathoracic cancer" comprises NLSC and distant recurrences without thoracic involvement.
d. Estimates derived with Fine–Gray methodology (cmprsk in R); values are percentages and may not sum exactly due to rounding.
e. Cohort size N=496; complete-case approach for time-to-event analyses.

© 2025 McMillan MT et al. *JAMA Network Open*.

eTable 3. Non-Lung Secondary Cancer (NLSC) Characteristics and Detection Mode Legend.
Site distribution and detection mode for NLSM events (N=39).

Cancer type, N (%)	Symptom-driven	Incidental	Total
All NLSC	27 (69.2)	12 (30.8)	39 (100)
Breast cancer	5 (12.8)	1 (2.6)	6 (15.4)
Prostate adenocarcinoma	5 (12.8)	0 (0)	5 (12.8)
Pancreatic ductal adenocarcinoma	3 (7.7)	2 (5.1)	5 (12.8)
Colon adenocarcinoma	2 (5.1)	1 (2.6)	3 (7.7)
HNSCC	5 (12.8)	0 (0)	5 (12.8)
Cutaneous melanoma	3 (7.7)	0 (0)	3 (7.7)
Urothelial carcinoma	2 (5.1)	1 (2.6)	3 (7.7)
Heme malignancy	0 (0)	3 (7.7)	3 (7.7)
Gynecologic cancer	0 (0)	2 (5.1)	2 (5.1)
Hepatocellular carcinoma	0 (0)	1 (2.6)	1 (2.6)
Gastrointestinal stromal tumor	1 (2.6)	0 (0)	1 (2.6)
Cutaneous squamous cell carcinoma	1 (2.6)	0 (0)	1 (2.6)
Renal cell carcinoma	0 (0)	1 (2.6)	1 (2.6)

Abbreviations. DCIS, ductal carcinoma in situ; HNSCC, head and neck squamous cell carcinoma; NLSC, non-lung secondary cancer.

a. Denominators for site-specific percentages are the total number of NLSC (N=39). "Total" equals symptom-driven + incidental.

b. Detection mode definitions: symptom-driven = evaluation prompted by new symptoms or abnormal examination; incidental = identified on tests or imaging obtained for other indications (including surveillance for the index lung cancer).

c. Stage at diagnosis refers to site-specific staging at the time of NLSC diagnosis; in situ includes DCIS for breast.

d. Screening status for cancers with established population screening (breast, colorectal, prostate) is summarized in the main text; all were up to date at diagnosis.

e. Categories "heme malignancy" and "HNSCC" encompass leukemia/lymphoma and head-and neck squamous cell carcinoma, respectively.

© 2025 McMillan MT et al. *JAMA Network Open*.

eTable 4. Cause-Specific Cox Regression for Risk of NLSC

Legend. Multivariable cause-specific Cox model estimating hazard ratios (HR) for incident NLSC; competing events (recurrence, second primary lung cancer, or death) are censored at their occurrence.

Characteristic	HR	95% CI	P value
Age	1.02	0.98-1.07	0.36
Pack years smoking (per 10)	0.88	0.77-1.00	0.05
AJCC Stage Group			
I	Ref		
II	0.85	0.29-2.53	0.77
III	0.37	0.09-1.45	0.15
Index NSCLC Histology			
Adenocarcinoma	Ref.		
Squamous cell carcinoma	1.41	0.54-3.69	0.49
Other	2.32	0.82-6.59	0.11
Prior cancer (ref=no)	0.94	0.45-1.97	0.87
RT for local therapy (ref=surgery)	1.44	0.45-4.60	0.54
Hereditary syndrome and/or pathogenic germline variant	8.32	3.14-22.02	<0.001
Male sex (reference=female)	1.35	0.68-2.68	0.39

Abbreviations. AJCC, American Joint Committee on Cancer; CI, confidence interval; HR, hazard ratio; NLSC, non-lung secondary cancer; NSCLC, non-small cell lung cancer; RT, radiotherapy.

a. Time zero: completion of definitive local therapy. Individuals experiencing competing events were censored at the event time.
b. Reference categories: AJCC stage I; index histology adenocarcinoma; no prior cancer; surgery for local therapy; female sex.
c. Continuous covariates: age per 1 year; pack-years per 10 (reported as “Pack years smoking [per 10]”).
d. Model fit on complete cases without imputation; 95% CIs are Wald-based; two-sided P values greater than or equal to 0.01 are reported to the nearest hundredth and P values less than 0.001 are reported as "<0.001”).

© 2025 McMillan MT et al. *JAMA Network Open*.
