# Supplementary material for: Risk and Outcomes of Secondary Cancer Among Lung Cancer Survivors After Definitive Treatment
Source: JAMA Netw Open. 2025 Dec 9;8(12):e2547831. doi: 10.1001/jamanetworkopen.2025.47831 (PMC12690424; doi:10.1001/jamanetworkopen.2025.47831)
Supplement: Supplement 2. — Data Sharing Statement [file jamanetwopen-e2547831-s002.pdf]

Data Sharing Statement

McMillan. Risk and Outcomes of Secondary Cancer Among Lung Cancer Survivors After Definitive Treatment. *JAMA Netw Open*. Published December 09, 2025. doi:10.1001/jamanetworkopen.2025.47831

Data
Data available: No

Additional Information
Explanation for why data not available: The data contain PHI.
